# Evaluation of index of cardiac-electrophysiological balance in patients with subarachnoid hemorrhage

**DOI:** 10.1186/s12872-022-02924-y

**Published:** 2022-11-10

**Authors:** Seyho Cem Yücetas, Hakan Kaya, Safiye Kafadar, Huseyin Kafadar, Hakan Tibilli, Ahmet Akcay

**Affiliations:** 1 Department of Neurosurgery, Adiyaman University Education and Research Hospital, Adiyaman, Turkey; 2 Department of cardiology, Adiyaman University Education and Research Hospital, Adiyaman, Turkey; 3 Department of Radiology, Adiyaman University Education and Research Hospital, Adiyaman, Turkey; 4 Department of Forensic Medicine, Harran University Research Hospital, Sanliurfa, Turkey

**Keywords:** Index of cardiac electrophysiological balance, Subarachnoid hemorrhage, Ventricular arrhythmia

## Abstract

**Background:**

Various electrocardiographic (ECG) changes occur after subarachnoid hemorrhage (SAH). Prolonged QT and corrected QT (QTc) intervals are notable changes. QT, QTc, T peak-to-end T(p-e) intervals, and Tp-e/QTc ratio are used as ventricular arrhythmia indices. In recent publications, the cardiac electrophysiological balance index (ICEB), which provides more information than other ECG parameters (QT, QTc, etc.), is recommended in predicting the risk of ventricular arrhythmia. This study aims to assess ICEB in aneurysmal SAH patients.

**Methods:**

The study included 50 patients diagnosed with aneurysmal SAH and 50 patients diagnosed with hypertension without end-organ damage as the control group. All patients’ Fisher scores and Glasgow Coma Scale (GCS) scores were recorded. Both groups were given 12-lead ECGs. QT, QTc, Tp-e intervals, QRS duration, ICEB (QT/QRS), ICEBc (QTc/QRS), and T(p-e)/QTc values were calculated and analyzed between groups.

**Results:**

Compared to the control group; QT (426,64 ± 14,62 vs. 348,84 ± 12,24 ms, p < 0,001), QTc (456,24 ± 28,84 vs. 392,48 ± 14,36 ms, p < 0,001), Tp-e (84,32 ± 3,46 vs. 70,12 ± 3,12, p < 0,001), Tp-e/QTc (0,185 ± 0,08 vs. 0,178 ± 0,02, p < 0,001), ICEB (4,53 ± 0,78 vs. 3,74 ± 0,28, p < 0,001) and ICEBc (4,86 ± 0,86 vs. 4,21 ± 0,24, p < 0,001) were significantly higher in patients with aneurysmal SAH. QT, QTc and Tp-e interval, Tp-e/QTc ratio, ICEB (QT/QRS) and ICEBc (QTc/QRS) were positively correlated with the Fisher score and were negatively correlated with the GCS. According to linear regression analyses, the ICEBc (QTc/QRS) found to be independently associated with the Fisher score.

**Conclusion:**

The values of the ICEB and ICEBc were significantly increased in patients with aneurysmal SAH. The severity of SAH was positively correlated with the ICEB and ICEBc. The ICEBc (QTc/QRS) independently associated with the Fisher score. This may that SAH suggest may predispose to malignant ventricular arrhythmias.

## Introduction

Cardiovascular complications are quite common in subarachnoid hemorrhage (SAH) patients. Various electrocardiographic (ECG) changes occur after SAH. Specifically, prolongation of QT and corrected QT (QTc) intervals has been demonstrated to be correlated with SAH which may cause severe ventricular arrhythmia [1]. It is considered that increased sympathetic activity and high catecholamine levels in SAH patients may cause QT and QTc prolongation [1]. SAH has a clinical spectrum ranging from minor symptoms such as headache to destructive and fatal consequences [2]. Early diagnosis of SAH is very important to prevent complications and death associated with SAH[3]. According to the international guidelines, all patients with intracranial hemorrhage should be evaluated during attendance by a standard 12-lead ECG to document any cardiac abnormality [4].

Index of cardiac electrophysiological balance (ICEB), a new index for predicting ventricular arrhythmia, is calculated by dividing QT interval to QRS duration (QT/QRS) [5]. ICEB reflects the balance between ventricular depolarization and repolarization. Wavelength λ which is an electrophysiological measurement is considered equivalent to ICEB (λ = effective refractory period× conduction velocity). ICEB have the advantages of being non-invasive and easy to be measured [6]. ICEB provides more information about the prediction of ventricular arrhythmia than the other ECG parameters (QT, QTc, etc.). To our knowledge, there has been no study in the literature using ICEB in order to predict ventricular arrhythmia in patients with SAH. This study aims to assess the value of ICEB in aneurysmal SAH patients.

## Material and method

### Study design

The present study was carried out at the Department of Neurosurgery and the Department of Cardiology. The study consisted of patients aged 18 years and older who developed the first acute aneurysmal SAH in less than 48 h from baseline. To grade SAH, Fisher scores and Glasgow Coma Scale were used (Table 1). All patients were performed emergency non-contrast CT brain scans at the emergency clinic. Radiologists evaluated the CT brain scans of all patients. Glasgow Coma Scale and Fisher scores were registered. Fifty patients with aneurysmal SAH participated in the study. Nimodipine was used in the treatment of these patients.

The control group comprise 50 patients aged 18 years and older, without end-organ damage, who admitted to the cardiology outpatient clinic due to hypertension. Approval was acquired from the local Ethics Committee. (The Ethics Committee, Adiyaman University, approval no: 2021/08–16). Informed consent was obtained from all subjects. The present study was done in accordance with the Helsinki Declaration. Blood pressure values and demographic data were obtained from those patients who accepted to participate in the study. Other variables measured including body mass index (BMI), smoking, creatine, blood sugar, electrolyte levels, alanine aminotransferase (ALT), thyroid-stimulating hormone, aspartate aminotransferase (AST), and blood samples were collected from the patients during their first visit to the clinic. Patients with rheumatic heart disease, systemic autoimmune disease, right or left branch block on their electrocardiography, valvular heart disease, thyroid dysfunction, liver disease, chronic lung disease, acute or chronic infection, electrolyte disorder, anemia, kidney disease, sinus arrhythmia in electrocardiography, atrioventricular conduction defect and those using antidepressants, antipsychotics, antiarrhythmic or antihistaminic drugs and those with stable or acute coronary arterial disease and diabetes were excluded from the study.

**Table 1:** Fisher score and Glasgow Coma Scale

Fisher Score	CT imaging	Glasgow Coma Scale
1	no bleeding	15-Asymptomatic, minimal headache, Mild neck stiffness, (no motor deficit)
2	< 1 mm bleeding	13-14- Middle-severe neck stiffness, no neurological deficit
3	≥ 1 mm bleeding	9-12-Mild neurological deficit, Confusion,
4	diffuse or intraventricular bleeding	3-8- Coma or deep coma

### Echocardiographic and electrocardiographic evaluation

The 12-lead ECG (Nihon Kohden, Tokyo, Japan) device was set at a rate of 50 mm/Sect. 1 mV. ECG was performed while the patients were in the supine position. We evaluated ECGs that were acquired at admission. All participants had sinus rhythm on ECG. Heart rate was calculated from the data obtained from the ECG along with T peak-to-end T(p-e) interval, QRS duration and QT distance. A magnifying glass was used to reduce the error rate while measuring. Lead V5 and Lead II leads were used for measurements. The part from the beginning of the QRS complex to the end of the T wave was determined as the QT interval. The QTc interval was recalculated according to heart rate using Bazett’s formula: QTc = QT√(R-R interval) [7]. Tp-e/QT, Tp-e/QTc, QT/QRS (ICEB) and QTc/QRS (ICEBc) values ​​were analyzed according to these measurements. Transthoracic echocardiography was performed with Vivid 5 (General Electric, Horten, Norway) device with 2,5 MHz transducer. The patients were tilted to the left and echocardiographic examination was performed. The patients were followed during the echocardiography procedure. Simpson’s method was used to calculate the left ventricular ejection fraction (LVEF) [8].

### Statistical analysis

Statistical Package for the Social Sciences version 22.0 (SPSS Inc., Chicago, IL, U.S.A.) was used for statistical analysis.

### Power analysis and sample size were made with G*power 3.1.9.2 program

According to the study of Avci et al. [9], when we assume that the mean QTc intervals of patients with subarachnoid hemorrhage were 510.48 (SD: 48.7), and those without subarachnoid hemorrhage were 392.8 (SD: 26.3), we calculated that a minimum of 10 patients were sufficient for the study at 0.05 of the alpha (α) error value and at 95% of the research power. As a result, we planned to recruit 50 patients for each group as dropouts may occur in the study.

Numerical variables were posted as mean ± standard deviation values, whereas qualitative variable were given as numbers and percentages. Independent sample t-tests were used to compare numerical variables, whereas Mann-Whitney U tests were used for discontinuous parametric variables. The Kolmogorov-Smirnov test was used to evaluate data distribution. Qualitative variables were compared by using chi-square tests within the study group. For correlation analysis, the Pearson correlation test was used. Linear regression analysis was used for the relationship between Glasgow Coma Scale, Fisher score and ECG parameters. It was considered statistically significant if the p values were less than 0.05.

## Results

This study included 50 patients with aneurysmal SAH (35 males, 15 females; mean age 53,84 ± 15,5 years) and 50 patients diagnosed with hypertension without end-organ damage (30 males and 20 females; mean age 57,34 ± 13 years). Echocardiographic findings, clinical features, and laboratory data of all patients are shown in Table 2. The clinical characteristics (age, gender, smoking status, BMI, Pulse oximeter, systolic and diastolic blood pressure) were found to be similar in both of groups. (p > 0,05, for all). The echocardiographic measurements (interventricular septum thickness, left ventricular ejection fraction, left ventricular posterior wall thickness) were similar in both groups, with no statistical difference (p > 0,05, for all). In terms of laboratory parameters (fasting glucose, creatinine, magnesium, potassium, calcium, thyroid-stimulating hormone, AST, and ALT), there were no significant differences between the two groups (p > 0,05, for all).

**Table 2:** Clinical characteristics, laboratory and echocardiographic findings of the groups

	Patients with SAH	Control group	p-value
	(n = 50)	(n = 50)	
Age (years)	53,84 ± 15,5	57,34 ± 13,7	0,342
Gender, male, n %	35 (70)	30(60)	0,412
BMI (kg/m²)	28,65 ± 2,45	26,75 ± 2,54	0,086
Smokers, n, (%) Pulse-oximeter (SaO2)	28(56) 96,84 ± 4,93	24(48) 98,02 ± 1,86	0,568 0,264
Systolic BP (mmHg)	172,68 ± 36,4	164,48 ± 40,56	0,486
Diastolic BP (mmHg)	96,52 ± 16,3	92,45 ± 14,8	0,536
IVS (mm)	11,58 ± 0,62	11,24 ± 0,58	0,482
PW (mm)	8,43 ± 1,16	8,12 ± 1,14	0,348
LVEF (%) AST (u/L) ALT (u/L	6043 ± 3,18 32,74 ± 14,28 30,56 ± 12,8	60,52 ± 2,94 30,26 ± 13,56 28,64 ± 16,48	0,214 0,084 0,054
Creatinine (mg/dL)	0,72 ± 0,22	0,6 ± 0,25	0,356
Potassium (mmol/L)	4,18 ± 0,56	4,23 ± 0,52	0,482
Calcium (mg/dL)	9,56 ± 0,35	9,65 ± 0,38	0,562
Magnesium (mg/dL)	2,03 ± 0,26	2,11 ± 0,24	0,228
Fasting glucose (mg/dL)	99,84 ± 12,82	95,68 ± 14,36	0,324
Thyroid stimulating hormone (µU/mL Glasgow coma scale Fisher score	2,48 ± 1,18 11,08 ± 4,03 3,18 ± 0,84	2,28 ± 1,26 15,00 ± 0,00 1,00 ± 0,00	0,086 < 0,001 < 0,001

ALT, alanine aminotransferase; AST, Aspartate aminotransferase; BMI, body mass index; BP, blood pressure; IVS, inter ventricular septum; PW, posterior wall; LVEF, left ventricle ejection fraction

The mean heart rate was similar between the control group and aneurysmal SAH patients (73,26 ± 12,36 beat/min and 72,18 ± 12,24 beat/min, respectively, p > 0,05) (Table 3). The QRS duration was also similar between the control group and aneurysmal SAH patients (93,16 ± 14,46 ms and 94,12 ± 20,86 ms, respectively, p > 0,05) (Table 3). Compared to the values of the control group, in the aneurysmal SAH patients, the QT duration (426,64 ± 14,62 ms vs. 348,84 ± 12,24 ms, p < 0,001), QTc (456,24 ± 28,84 vs. 392,48 ± 14,36 ms, p < 0,001), Tp-e (84,32 ± 3,46 vs. 70,12 ± 3,12, p < 0,001), Tp-e/QTc (0,185 ± 0,08 vs. 0,178 ± 0,02, p < 0,001), ICEB (4,53 ± 0,78 vs. 3,74 ± 0,28, p < 0,001) and ICEBc (4,86 ± 0,86 vs. 4,21 ± 0,24, p < 0,001) were high in a statistically significant way. (Table 3). QT interval, QTc interval, Tp-e interval, Tp-e/QTc ratio, ICEB (QT/QRS) and ICEBc (QTc/QRS) were positively correlated with the Fisher score and were negatively correlated with the Glasgow Coma Scale score (Table 4). According to linear regression analyses, the ICEBc (QTc/QRS) found to be independently associated with the Fisher score (Table 5).

**Table 3:** Electrocardiographic findings of the groups

	Patients with SAH	Control group	p-value
	(n = 50)	(n = 50)	
Heart rate (beats/min)	72,18 ± 12,24	73,26 ± 12,36	0,658
QRS (ms)	94,12 ± 20,86	93,16 ± 14,46	0,726
QT interval (ms)	426,64 ± 14,62	348,84 ± 12,24	< 0,001
QTc interval (ms)	456,24 ± 28,84	392,48 ± 14,36	< 0,001
Tp-e interval (ms)	84,32 ± 3,46	70,12 ± 3,12	< 0,001
Tp-e/QTc ratio	0,185 ± 0,08	0,178 ± 0,02	< 0,001
ICEB (QT/QRS)	4,53 ± 0,78	3,74 ± 0,28	< 0,001
ICEBc (QTc/QRS)	4,86 ± 0,86	4,21 ± 0,24	< 0,001

QTc, corrected QT interval; Tp-e, T wave peak-to-end; ICEB, index of cardio-electrophysiological balance; ICEBc, corrected ICEB

**Table 4:** Correlation Analysis of QT interval, QTc interval, Tp-e interval, Tp-e/QTc ratio, ICEB (QT/QRS) and ICEBc (QTc/QRS) with GCS and Fisher score in patients with subarachnoid hemorrhage

		GCS	Fisher score
**QT interval**	r	-0.536	0.645
	p	< 0.001	< 0.001
**QTc interval**	r	-0.532	0.735
	p	< 0.001	< 0.001
**Tp-e interval**	r	-0.558	0.845
	p	< 0.001	< 0.001
**Tp-e/QTc ratio**	r	-0.521	0.813
	p	< 0.001	< 0.001
**ICEB (QT/QRS)**	r	-0.525	0.675
	p	< 0.001	< 0.001
**ICEBc (QTc/QRS)**	r	-0.540	0.745
	p	< 0.001	< 0.001

GCS, Glasgow coma scale; QTc, corrected QT interval; Tp-e, T wave peak-to-end; ICEB, index of cardio-electrophysiological balance; ICEBc, corrected ICEB

**Table 5:** Linear regression analysis for parameters significantly correlated with GCS and Fisher score

		GCS	Fisher score
**QT interval**	ß	-0.126	0.378
	p	0.256	0.064
**QTc interval**	ß	-0.108	0.402
	p	0.348	0.054
**Tp-e interval**	ß	-0.032	0.306
	p	0.749	0.102
**Tp-e/QTc ratio**	ß	-0.013	0.412
	p	0.912	0.051
**ICEB (QT/QRS)**	ß	-0.114	0.350
	p	0.312	0.086
**ICEBc (QTc/QRS)**	ß	-0.104	0.736
	p	0.362	< 0.001

GCS, Glasgow coma scale; QTc, corrected QT interval; Tp-e, T wave peak-to-end; ICEB, index of cardio-electrophysiological balance; ICEBc, corrected ICEB. R square for QT; QTc, Tp-e interval; Tp-e/QTc ratio; ICEB and ICEBc as 648, 654, 784, 708, 624 and 632 respectively

## Discussion

Our study is the first to show the increase in ICEB and ICEBc values in aneurysmal SAH patients. A positive correlation between ICEB, ICEBc and SAH severity has been established. In addition a linear regression analyses showed the ICEBc (QTc/QRS) independently associated with the Fisher score. Intracranial lesions have severe cardiovascular effects, and these effects are associated with increased mortality and morbidity [10]. Various ECG changes occur in patients with SAH. Among these changes, QT and QTc prolongation are the most important. QT and QTc prolongation play a unique role because they both include ventricular depolarization and repolarization periods, and its prolongation may predict the risk for malignant ventricular arrhythmias [11]. QT prolongation is commonly observed in SAH patients with fatal ventricular arrhythmias like Torsades de Pointes [12, 13]. In our study, we have observed that QT and QTc intervals are prolonged in aneurysmal SAH patients.

The electrocardiographic T wave reflects ventricular repolarization, and the interval between the peak and the end of a single T wave is defined as the Tp-e interval. The new parameters predicting ventricular repolarization dispersion are Tp-e interval, Tp-e/QT, and Tp-e/QTc ratios [14, 15]. Prolonged Tp-e interval is independently associated with ventricular arrhythmia and sudden cardiac death and ıt is useful when the QT is regular or cannot be measured due to a prolonged QRS duration [16]. Recently, it has been claimed that the Tp-e/QT ratio may be used as an accurate index for the distribution of ventricular repolarization independent of heart rate changes [16]. Further, it has been asserted that the Tp-e/QT ratio is more accurate than Tp-e, QT, and QTc intervals in predicting ventricular arrhythmia [17]. It has been shown that the Tp-e interval and Tp-e/QT ratio increase in heart diseases such as Brugada syndrome, short QT syndrome, long QT syndrome, acute myocardial infarction and also, ın some studies, it has been found that the Tp-e interval and Tp-e/QT ratio increase in non-cardiac diseases such as autoimmune hepatitis, obstructive sleep apnea and psoriasis [18–21]. These studies emphasized that increased Tp-e interval and Tp-e/QT ratio might be associated with increased ventricular arrhythmias. There is only one study evaluating the Tp-e interval and Tp-e/QTc ratio in SAH patients in the literature [9]. This study showed that the Tp-e interval and Tp-e/QTc ratio were increased in nontraumatic SAH patients. Similarly, the Tp-e interval and Tp-e/QTc ratio in our study were significantly higher in aneurysmal SAH patients.

ICEB is a non-invasive parameter that shows ventricular proarrhythmic risk and may provide information about the cardiac action potential depolarization and repolarization phases. Thanks to this characteristic, ICEB can better predict the cardiac proarrhythmic risk than Tp-e interval, Tp-e/QT ratio and QT interval instability showing only repolarization [5]. High ICEB values are associated with Torsades de Pointes, whereas low values are associated with non-Torsades de Pointes mediated ventricular arrhythmias [5]. There are few studies on ICEB in the literature. ICEB value is increased in cardiac diseases such as increased pericardial volume or acute myocarditis with arrhythmia and non-cardiac diseases such as rheumatoid arthritis, tinnitus, and end-stage renal disease [22–26]. Our study observed that the ICEB and ICEBc values were significantly high in aneurysmal SAH patients. Further, a positive correlation between ICEBc and SAH severity has been shown in our study. High ICEB values ​​in aneurysmal SAH patients may be due to autonomic nervous system disorders and increased sympathetic nervous system activity. The autonomic nervous system plays a vital role in the occurrence of ventricular arrhythmias, as it is the most important regulator of ventricular repolarization [27]. Some mechanisms linking autonomic nervous system dysfunction and ventricular arrhythmias are well known [28]. Previous studies have claimed that dysfunction in the sympathetic and parasympathetic nervous systems causes a cardiac arrhythmia, affecting ventricular repolarization [29, 30]. Another reason for high ICEB values may be catecholamines, which increase in parallel to the increases in intracranial pressure. The correlation between serum catecholamine levels and ECG changes remains unclarified. The results of a study have shown that post-SAH high catecholamine levels do not play a direct role in the pathogenesis of long-term ECG changes [31]. However, researchers have demonstrated that experimental adrenaline infusion in serum causes prolonged QTc intervals in regular volunteers [32]. Increased QTc interval may cause high ICEB value in aneurysmal SAH patients. iCEB has some degree of heart-rate dependency, meaning that iCEB, like the QT interval itself, should optimally be corrected for underlying heart rate. In a previous study, Dabrowski et al. observed that ICEB values ​​were significantly higher in traumatic brain injury patients who underwent decompressive craniectomy who especially developed cardiac arrhythmia [33]. They suggested that the iCEB was relatively increased before decompressive craniectomy in patients who eventually experienced cardiac arrhythmias after decompressive craniectomy. Dabrowski et al. stated that not calculating the ICEBc was an important limitation of the their study, and also emphasized that calculating corrected ICEBc would be more accurate in future studies. In our study, we evaluated ICEBc values ​​as well as ICEB values. We observed a positive correlation between ICEBc and SAH severity.

## Study limitations

The present study has some limitations. The small number of patients, the use of single hospital data in the study, and the lack of follow-up of patients in terms of malignant ventricular arrhythmias or sudden cardiac death are the main limitations of this study.

## Conclusion

In conclusion, our study has demonstrated that iCEB and iCEBc values were statistically higher in aneurysmal SAH patients. Further, a positive correlation between ICEBc and SAH severity has been shown. Our results suggest that SAH may create a predisposition to malignant ventricular arrhythmias. Our results require confirmation by advanced multicenter studies in which patients are followed up for ventricular arrhythmia.

## Data Availability

The datasets used and analyzed during the current study are available from the corresponding author on reasonable request.

## Abbreviations

SAH: Subarachnoid hemorrhage
ECG: Electrocardiographic
ICEB: Index of cardiac electrophysiological balance
GCS: Glasgow Coma Scale
BMI: Body mass index
ALT: Alanine aminotransferase
AST: Aspartate aminotransferase
LVEF: Left ventricular ejection fraction
QTc: Corrected QT
T(p-e): T peak-to-end.
